# Research on Low-Profile Directional Flexible Antenna with 3D Coplanar Waveguide for Partial Discharge Detection

**DOI:** 10.3390/mi16030253

**Published:** 2025-02-24

**Authors:** Yan Mi, Wentao Liu, Yiqin Peng, Lei Deng, Benxiang Shu, Xiaopeng Wang, Songyuan Li

**Affiliations:** 1 State Key Laboratory of Power Transmission Equipment Technology, School of Electrical Engineering, Chongqing University, Chongqing 400044, China; liuwentao@stu.cqu.edu.cn (W.L.); pyq@stu.cqu.edu.cn (Y.P.); dl@stu.cqu.edu.cn (L.D.); 202411131288@stu.cqu.edu.cn (B.S.); 2 State Grid Tianjin High Voltage Company, Tianjin 300000, China; wxp339@163.com; 3 State Grid Tianjin Electric Power Research Institute, Tianjin 300384, China; lisongyuan1987@163.com

**Keywords:** GIS cable terminal, partial discharge, flexible antenna, ultra-high-frequency (UHF) method

## Abstract

Due to the challenges in antenna installation and detection performance caused by metal obstruction along the propagation path at a Gas-Insulated Switchgear (GIS) cable terminal, as well as the adverse effects of environmental interference on the detection of partial discharge (PD) by existing flexible antennas, this paper proposes a directional flexible antenna design to mitigate these issues and improve detection performance. The proposed design employs a coplanar waveguide (CPW)-fed monopole antenna structure, where the grounding plane is extended to the back of the antenna to enhance directional reception. The designed flexible antenna measures 88.5 × 70 × 20 mm, and its low-profile design allows it to be easily mounted on the outer wall of the epoxy sleeve at the GIS cable terminal. The measurement results show that the flexible antenna has a Voltage Standing Wave Ratio (VSWR) of less than 2 in the 0.541–3 GHz frequency range. It also maintains stable impedance characteristics across various bending radii, with an average effective height of 10.79 mm in the 0.3–1.5 GHz frequency range. A GIS cable terminal PD experimental platform was established, and the experimental results demonstrate that the bending has minimal impact on the detection performance of the flexible antenna, which can cover the detection range of the GIS cable terminal; metal obstruction significantly impacts the PD signal amplitude, and the designed flexible antenna is suitable for detecting PDs in confined spaces with metal obstruction.

## 1. Introduction

GIS is widely used in power systems due to its high operational stability, compact size, and strong resistance to external interference [1,2]. However, once an insulation fault occurs within GIS, the difficulty and complexity of repair exceed those of conventional electrical equipment. Research has shown that insulation degradation caused by PD is a critical factor in GIS insulation degradation [3,4,5]. When PD occurs inside GIS, electromagnetic signals in the UHF band (0.3–3 GHz) are generated [6]. The UHF method detects these signals using antennas, thereby reflecting the associated information and enabling online monitoring of PD in GIS. This method demonstrates advantages such as high anti-interference capability, operational flexibility, and non-contact functionality, establishing it as a focal point of interest for numerous researchers.

External antennas used for PD detection in GIS are typically installed on structures that allow electromagnetic waves to pass through, such as bell-type insulators, flange gaps, and cable terminal [7,8,9]. Among these, the cable terminal serves as an intermediate structure connecting external cables to the GIS chamber. The electric field distribution in these areas is non-uniform, increasing the likelihood of faults and PD. When PD occurs, the UHF signals in the chamber are radiated outward through the cable terminal, making it an ideal monitoring point for GIS PD detection. Reference [10] designed a compact log-periodic planar dipole antenna with dimensions of 140 × 110 × 3.2 mm, which covers a frequency range of 0.8–2.2 GHz. Reference [11] proposed a composite PD flexible antenna consisting of an equiangular spiral and an Archimedean spiral, with a diameter of 140mm and a thickness of 0.3 mm, covering a bandwidth of 0.44–2.98 GHz, and maintaining stable characteristics after multiple bending deformations. Reference [12] studied a 3D helical antenna with a flexible feed network to improve the signal-to-noise ratio (SNR) and reduce antenna size, with an average gain greater than 5 dB in the 0.5–2 GHz band, which effectively detects PD signals. However, due to the compact installation space of the cable terminal, which is obstructed by metal components such as screws, existing PD detection antennas are often rigid and challenging to install in confined and complex spaces, affecting detection performance. Some scholars have explored the flexibility of antennas, but most studies on flexible PD detection antennas focus on internal designs [13,14,15,16], without considering the impact of external interference on detection.

To address the aforementioned issues, this paper first designs and simulates a flexible antenna, then fabricates and measures the designed antenna, and finally conducts PD tests using the flexible antenna. The specific content of each section is as follows: in Section 2, based on the design principles of the CPW-fed monopole antenna and considering on-site installation conditions, the CPW ground plane is extended to the back of the antenna to develop a low-profile three-dimensional flexible antenna with directional signal reception capability. The antenna utilizes flexible polyimide (PI) as the substrate material, and HFSS is employed to establish a physical model for multi-parameter optimization concerning shape, dimensions, and VSWR. In Section 3, the previously designed flexible antenna is fabricated and subjected to experimental validation. A vector network analyzer and a GTEM chamber are used to measure its VSWR and effective height. In Section 4, a simulated GIS cable terminal partial discharge test platform is constructed. The designed flexible antenna is tested under different bending conditions, varying detection distances, and different levels of metal obstruction. Additionally, a comparative analysis of the detection performance between the flexible antenna and an existing finished antenna is conducted. In Section 5, the research findings are summarized, and the advantages of the flexible antenna designed in this paper are highlighted.

## 2. Flexible Antenna Design

### 2.1. Design Principle of CPW-Fed Monopole Antenna

**Figure 1:**
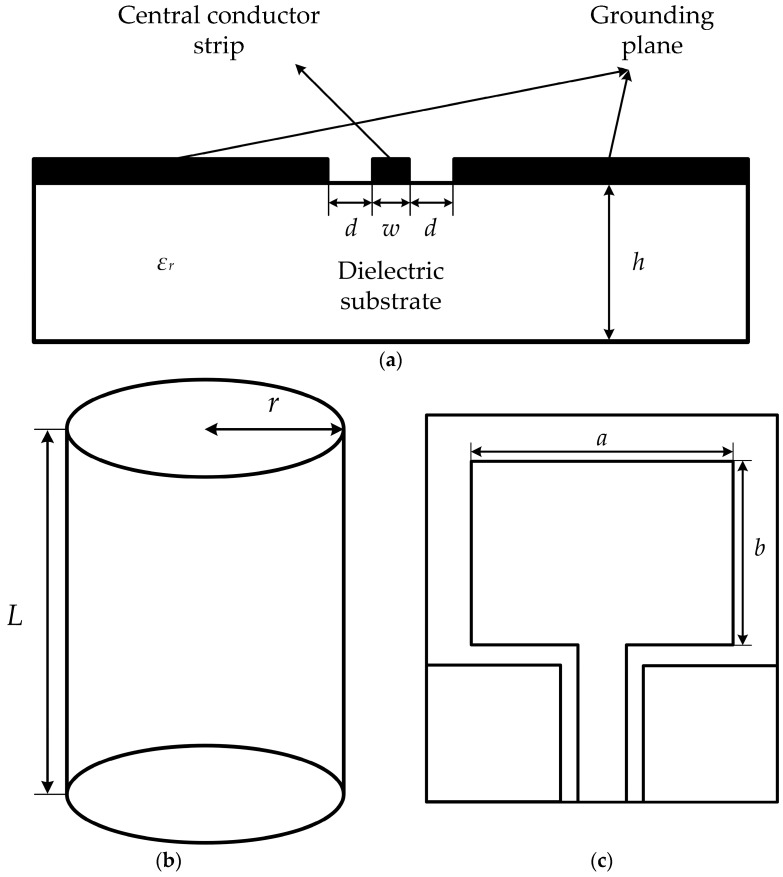
Basic antenna structure. (**a**) CPW structure schematic; (**b**) cylindrical radiating element; (**c**) planar monopole antenna.

The CPW structure was first proposed by Professor C.P. Wen in 1969 [17]. The structure is shown in Figure 1a and consists mainly of a dielectric substrate with a central conductor band and ground planes on the surface. Due to its low parasitic parameters, good dispersion properties, suitability for broadband applications, and low radiation loss [18,19], the CPW structure has received significant attention and is widely used in antenna designs.

The impedance of a CPW structure can be derived using the conformal transformation method:

(1) $$Z_{0}=\frac{Z_{01}}{\sqrt{\varepsilon_{e}}},$$

where *ε_e_* is the effective dielectric constant of the coplanar waveguide (CPW) and *Z*_01_ is the characteristic impedance of the CPW when *ε_r_* = 1, and the calculation method for both is as follows:

The effective dielectric constant *ε_e_* can be determined using the quasi-static method:

(2) $$\begin{array}{l} \varepsilon_{e}=\frac{\varepsilon_{r}+1}{2}\left\{\tan\left[0.775\ln\left(\frac{h}{d}\right)+1.75\right]\right.\\ \;\;\;\;\;\left.+\frac{kd}{h}\left[0.04-0.7k+0.01\left(1-0.01\varepsilon_{r}\right)\left(0.25+k\right)\right]\right\} \end{array},$$

where *k* = *w*/(*w* + 2*d*); *d* is the gap width between the central conductor strip and the grounding plane; *h* is the thickness of the dielectric substrate; and *w* is the width of the central conductor strip.

(3) $$Z_{01}=\frac{1}{4c\varepsilon_{0}}\frac{K^{\prime }\left(k\right)}{K\left(k\right)},$$

where *c* is the speed of light in a vacuum; *ε*_0_ is the dielectric constant of air; *K*(*k*) is the complete elliptic integral of the first kind; and *K’*(*k*) = *K*(*k’*), *k’* = (1 − *k*^2^)^1/2^.

The approximate calculation formula for *K’*(*k*)/*K*(*k*) is as follows:

(4) $$\frac{K^{\prime }\left(k\right)}{K\left(k\right)}=\left\{\begin{array}{cc} \frac{1}{\pi }\ln\left(2\frac{1+\sqrt{k^{\prime }}}{1-\sqrt{k^{\prime }}}\right) & \left(0\leq k\leq 0.7\right)\\ {\left[\frac{1}{\pi }\ln\left(2\frac{1+\sqrt{k^{\prime }}}{1-\sqrt{k^{\prime }}}\right)\right]}^{-1} & \left(0.7\leq k\leq 1\right) \end{array}\right.,$$

Using the above formulas, the characteristic impedance of the CPW can be controlled to approximately 50 Ω during the design process, allowing for signal transmission matching with coaxial cable impedance.

The planar monopole antenna has the advantages of a low profile and omnidirectional radiation. When combined with a CPW feeding structure, it has broad applications in short-range wireless communication systems.

The structure of a CPW-fed monopole antenna is shown in Figure 1c, where the radiating patch of the monopole antenna can be equivalently derived from the cylindrical radiating element shown in Figure 1b.

According to the study in [20], the side area of the cylindrical radiating element is equal to the area of the monopole patch:

(5) 2πrL=ab,

where *r* represents the radius of the cylindrical radiating element; *L* denotes the length of the cylindrical radiating element; *a* is the length of the monopole radiating patch; and *b* is the width of the monopole radiating patch.

Additionally, according to the aforementioned reference, for the cylindrical radiating element, we have

(6) $$\left\{\begin{array}{c} L=0.24\lambda F\\ F=\frac{L}{L+r}\\ f=\frac{c}{\lambda } \end{array}\right.,$$

where *λ* is the wavelength corresponding to the operating frequency; *F* is the degree of widening of the cylindrical radiating element; *f* is the operating frequency; and *c* is the speed of light. This equation reveals the relationship between the antenna’s geometric parameters and its operating frequency.

### 2.2. Flexible Antenna Design Concept and Structure

**Figure 2:**
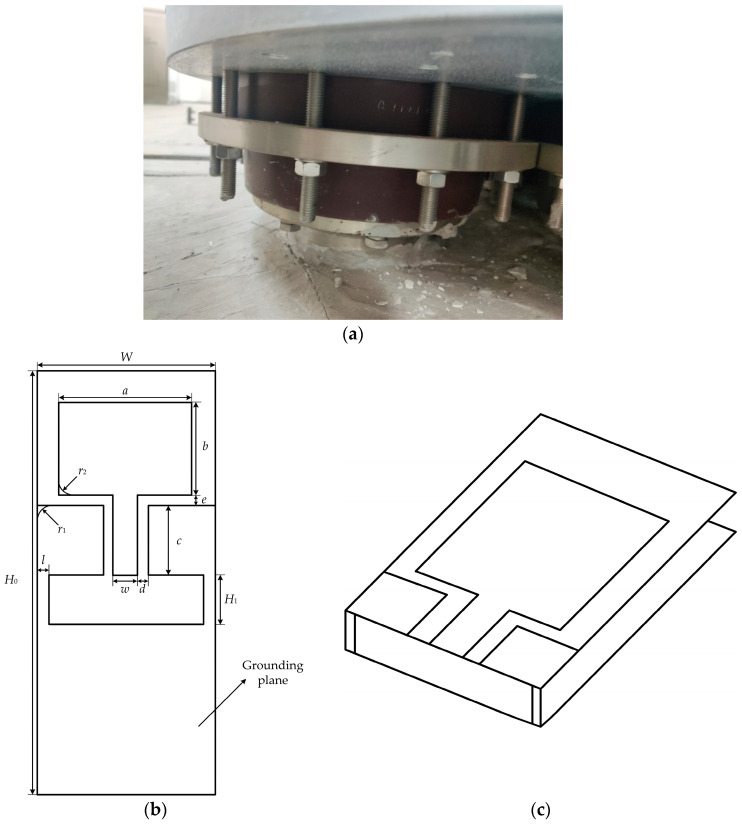
GIS cable terminal and antenna structure schematic. (**a**) GIS cable terminal; (**b**) unfolded antenna diagram; (**c**) 3D coplanar waveguide antenna.

Due to the structural characteristics of the GIS cable terminal, most of the external space is enclosed by the GIS shell, leaving only a small non-metallic structure around the epoxy sleeve of the cable terminal. Therefore, when PD occurs in the cable terminal, the UHF PD signals will propagate outward from the epoxy sleeve structure. This structure becomes a favorable window for detecting PD in GIS cable terminal [21]. As shown in Figure 2a, the epoxy structure around the cable terminal is often surrounded by metallic components such as flanges and screws. These metallic components hinder the propagation of the UHF electromagnetic waves generated by PD, which can affect the detection performance of UHF antennas. On the other hand, the distance between the metal screws and the epoxy sleeve is very small, typically only 2–3 cm. This makes it impossible for existing rigid ultra-high-frequency (UHF) antennas to be installed and used for detection within the limited space, especially in the presence of an electromagnetic shielding enclosure.

In order to install the antenna effectively on the outer wall of the epoxy sleeve of the GIS cable terminal for online PD monitoring, while minimizing interference from external signals, this paper designs a low-profile 3D flexible antenna based on the CPW-fed monopole antenna. The CPW grounding plane is extended to the back of the antenna and connected into a single structure, as shown in Figure 2b,c. This design enables the antenna to achieve directional signal reception, thereby reducing the reception of interference from the primary direction of incoming signals in the installation environment of the GIS cable terminal.

Due to the structural limitations of the GIS cable terminal, traditional rigid antennas are difficult to install effectively for PD detection. Therefore, compared to using rigid dielectric substrate antennas, a flexible dielectric substrate antenna that can conform to the outer wall of the epoxy sleeve achieves superior performance in terms of conformal detection [22,23]. The commonly used flexible dielectric substrate materials for antennas are shown in the following Table 1:

In general, the relative dielectric constant and the loss tangent of the dielectric substrate affect the signal delay and loss of the antenna [24]. Materials with a smaller loss tangent are typically chosen, while the relative dielectric constant should not be too low, as this would hinder broadband operation. Based on these considerations, PI is used as the dielectric substrate material for the antenna, and copper with a thickness of 0.035 mm is selected for the antenna conductors.

### 2.3. Low-Profile Directional Flexible Ultra-Wideband Antenna Optimization and Simulation

The shape parameters of the antenna affect its impedance matching as well as signal radiation and reception capabilities. Based on the earlier antenna size calculation formulas and the constraints of the GIS cable terminal dimensions, preliminary shape parameters for the flexible antenna were determined, as shown in Table 2. To achieve better antenna performance, the remaining shape parameters were discussed and optimized based on the antenna structure shown in Figure 2b.

**Figure 3:**
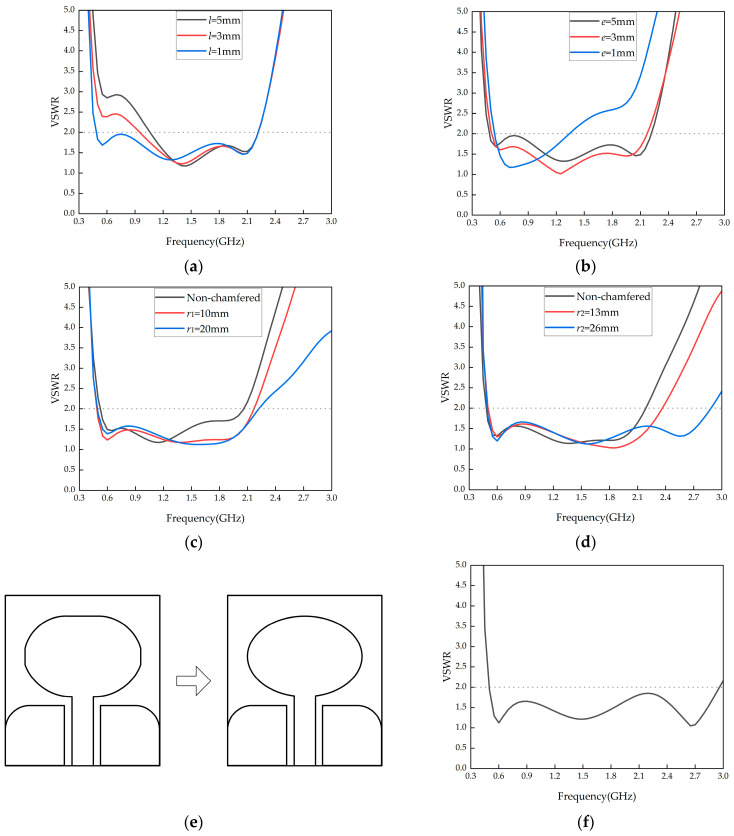
VSWR simulation optimization curves. (**a**) *l* parameter optimization simulation result; (**b**) *e* parameter optimization simulation result; (**c**) *r*_1_ parameter optimization simulation result; (**d**) *r*_2_ parameter optimization simulation result; (**e**) evolution of the monopole shape; (**f**) optimized antenna VSWR simulation curve.

Since the grounding plane is bent to the back of the antenna to adjust its signal reception, the conductor width *l* that connects the upper and lower grounding planes on the antenna’s side will affect the antenna’s VSWR. Simulations were performed by adjusting *l*, and the results are shown in Figure 3a. The simulation results indicate that as *l* decreases, the impedance matching of the antenna in the low-frequency range improves. Considering antenna processing and material strength, *l* = 1 mm is chosen.

The distance *e* between the grounding plane and the antenna patch affects the capacitive component of the antenna’s impedance. When *e* is too small, the capacitance increases, which deteriorates impedance matching. Thus, the value of *e* is adjusted for better impedance matching. The simulation results for varying e are shown in Figure 3b. The results show that reducing *e* improves low-frequency impedance matching, but as *e* decreases further, the high-frequency matching becomes worse. To balance low- and high-frequency impedance matching, *e* = 2 mm is chosen.

Sharp edges introduce parasitic capacitance and inductance, affecting the continuity of the antenna’s impedance. Therefore, the grounding plane and the antenna patch are rounded for optimization. The simulation results for rounding the grounding plane are shown in Figure 3c. As the rounding radius increases, the antenna bandwidth shifts towards higher frequencies. However, if the rounding radius is too large, the low-frequency impedance matching deteriorates. A compromise radius of *r*_1_ = 13 mm is selected.

The simulation results for rounding the antenna patch are shown in Figure 3d. As the rounding radius increases, the VSWR in the high-frequency range improves significantly. When *r*_2_ = 26 mm, the antenna patch becomes elliptical. Therefore, the rectangular antenna patch is optimized into an ellipse with a long axis of 34.5 mm and a short axis of 26.5 mm, and the schematic of the monopole transitioning from a rounded chamfer into an elliptical shape is shown in Figure 3e. The final shape parameters of the antenna are listed in Table 3, and the antenna’s optimized VSWR is shown in Figure 3f.

**Figure 4:**
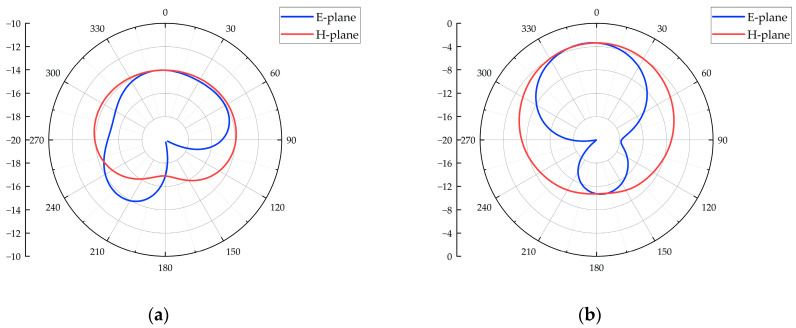

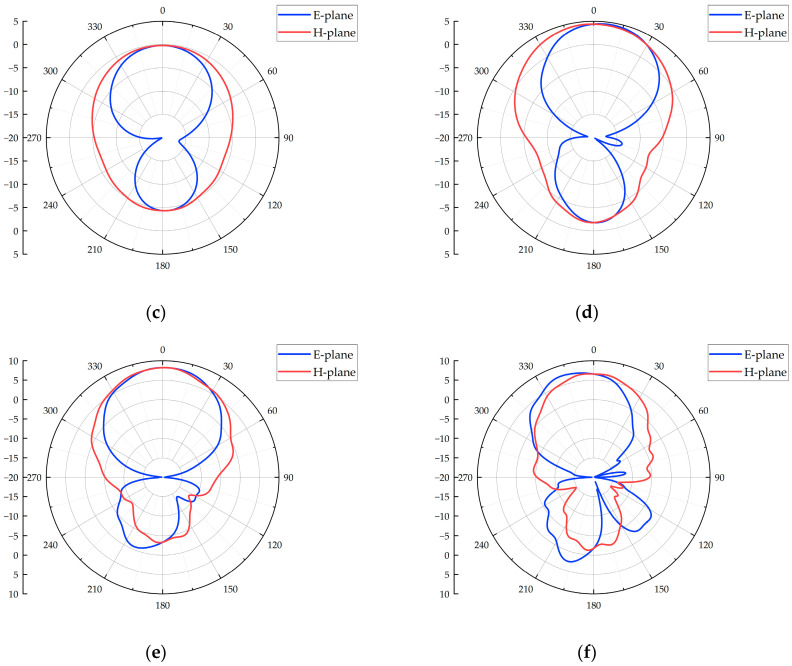
Two-dimensional radiation patterns of the flexible antenna: (**a**) 0.5 GHz; (**b**) 1.0 GHz; (**c**) 1.5 GHz; (**d**) 2.0 GHz; (**e**) 2.5 GHz; (**f**) 3.0 GHz.

The 2D radiation patterns of the antenna reflect its ability to radiate or receive signals in specific planes. Figure 4 shows the E-plane and H-plane 2D radiation patterns of the flexible antenna at different frequencies. From the figure, it can be observed that the grounding plane on the back of the 3D flexible antenna suppresses its radiation or reception capabilities to some extent. Moreover, as the frequency increases, the gain difference between the front and back of the antenna gradually increases. This suppression of noise signals from the back of the antenna helps reduce interference during PD detection. Sidelobes appear in the radiation pattern when the frequency exceeds 2 GHz, likely due to higher electromagnetic wave modes being excited by the increased frequency. However, the overall pattern remains within acceptable design limits and has minimal impact on the actual PD detection.

## 3. Low-Profile 3D Flexible Antenna Performance Testing

### 3.1. Flexible Antenna VSWR Testing

**Figure 5:**
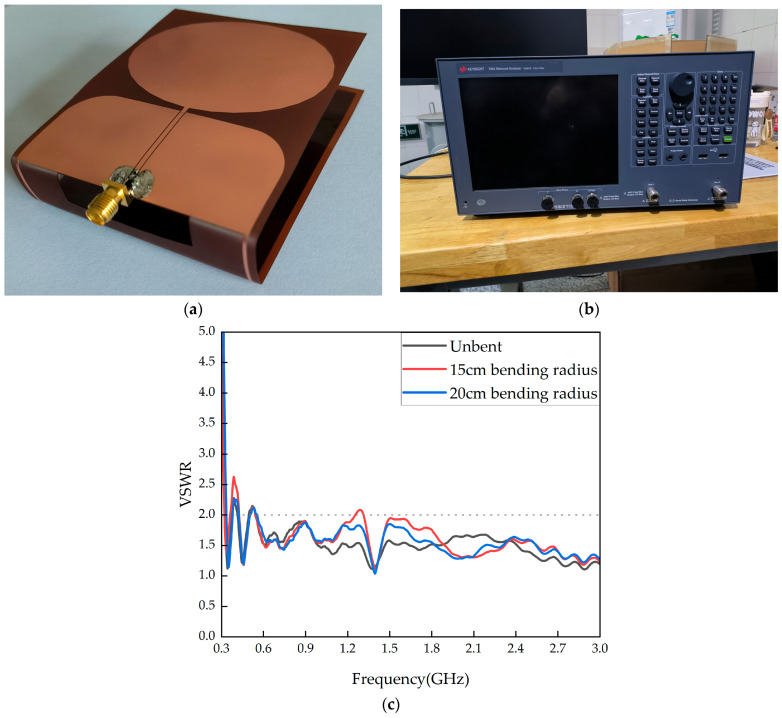
Measured VSWR of flexible antenna. (**a**) Physical prototype of the flexible antenna; (**b**) vector network analyzer; (**c**) measured VSWR curve of the flexible antenna.

The fabricated prototype of the flexible antenna, designed based on the previous section, is shown in Figure 5a. The processed flexible antenna was measured using a KEYSIGHT E5061B vector network analyzer, as shown in Figure 5b, and the measured results are presented in Figure 5c. The measurements indicate that the flexible antenna, under unbent conditions, achieves a VSWR ≤ 2 within the frequency range of 0.541–3 GHz. Comparing the simulated and measured VSWR, the overall bandwidth difference is less than 1%, with the measured bandwidth slightly shifting towards the higher frequency band. This is primarily due to errors from SMA (SubMiniature version A) connector soldering and processing during antenna fabrication, which resulted in slight frequency deviations. However, overall, the antenna still meets the design specifications.

As the flexible antenna is installed in the GIS cable terminal with varying bending radii, its VSWR was measured at different bending radii for comparison. Based on the measured results, the flexible antenna achieves a VSWR ≤ 2 in the 0.543–1.254 GHz and 1.313–3 GHz frequency ranges under a bending radius of 15 cm and within 0.554–3 GHz under a bending radius of 20 cm. As the bending degree increases, the impedance matching degrades slightly, but the overall matching remains good. The bandwidth is not significantly affected, with the overall bandwidth change being less than 1%. This shows that the designed flexible antenna can maintain stable impedance characteristics across different installation environments.

### 3.2. Flexible Antenna Effective Height Testing

The effective height represents the ability of the antenna to convert received electromagnetic signals into output voltage within a specific frequency band. Its expression is as follows:

(7) $$H_{e}\left(f\right)=\frac{U_{o}\left(f\right)}{E_{i}\left(f\right)},$$

where *f* is the signal frequency; *E*_i_(*f*) is the electric field strength amplitude of the incident electromagnetic wave at frequency *f*; and *U_o_*(*f*) is the output voltage amplitude at frequency *f*.

**Figure 6:**
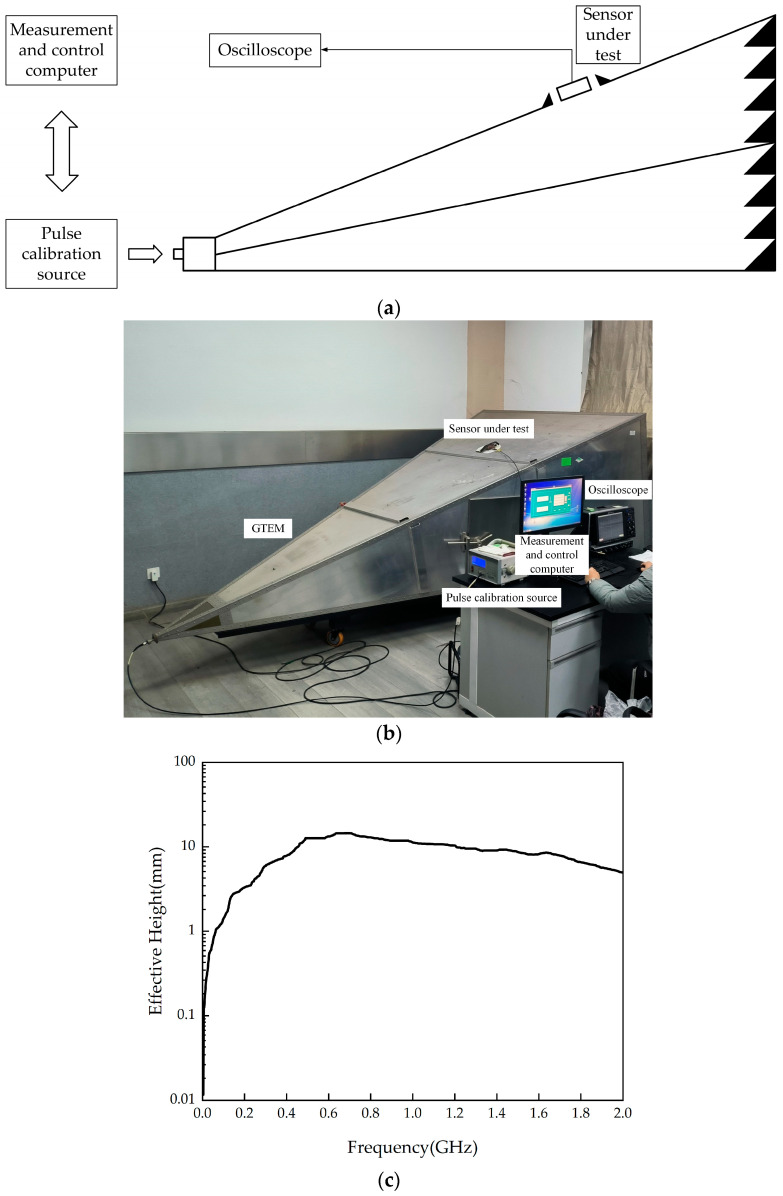
Effective height experimental platform and measurement curve. (**a**) GTEM experimental measurement system; (**b**) GTEM test system physical setup; (**c**) measured effective height curve of the flexible antenna.

To measure the effective height, a Gigahertz Transverse Electro-Magnetic Cell (GTEM), shown in Figure 6a,b, was used. The results are shown in Figure 6c. The measured data indicate that, in the 0.3–1.5 GHz frequency range, the average effective height of the flexible antenna is 10.79 mm, which meets the requirements for PD detection [25].

## 4. Flexible Antenna PD Detection Experiment

### 4.1. PD Experiment Platform

To verify the detection performance of the flexible antenna for PD (PD) signals, an experimental platform simulating the GIS cable terminal environment was set up in the laboratory. This platform mainly targets metal obstruction, such as screws and flanges, shown in Figure 2a, to simulate the constrained installation environment and reflect the impact of metal obstruction on PD detection results.

**Figure 7:**
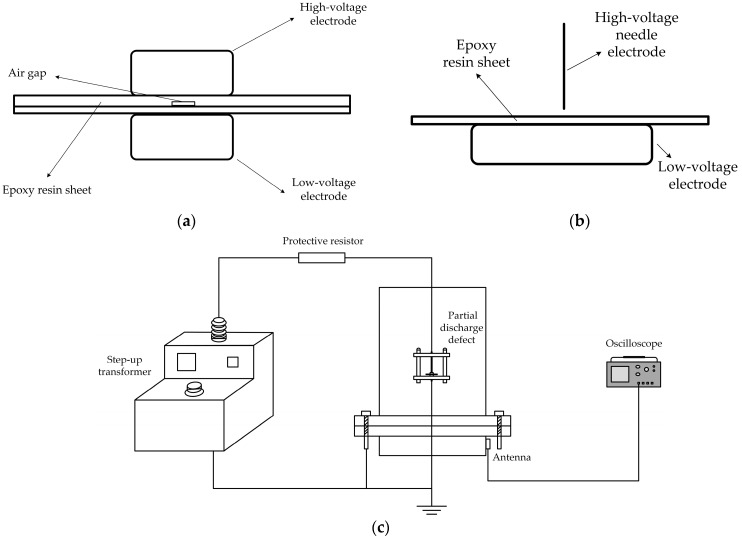

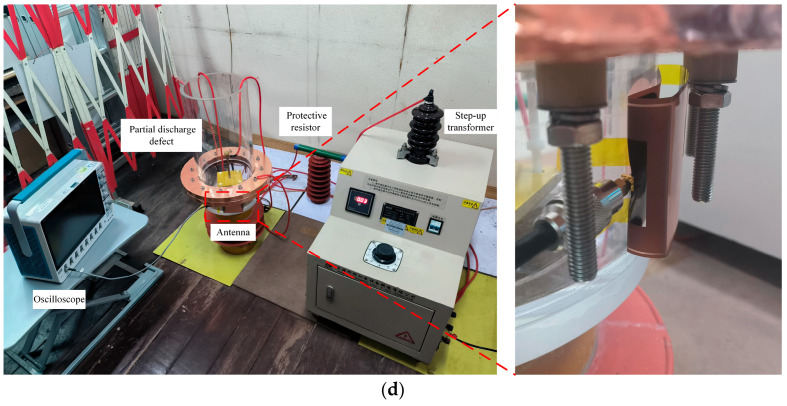
PD experiment platform. (**a**) Typical air gap defect model; (**b**) typical metal protrusion defect model; (**c**) experimental circuit schematic; (**d**) PD experimental platform.

For a cable terminal, installation quality and product defects are the main factors leading to faults. For example, defects such as rough surfaces, scratches, or internal air gaps in the insulation during manufacturing can lead to PD. Two typical PD defect types were selected as experimental models: air gap defects and metal protrusion defects. The defect setups are shown in Figure 7a,b. In the air gap defect, the total thickness of the two epoxy resin layers is 2 mm, with an air gap height of 0.5 mm. In the metal protrusion defect, the angle of the high-voltage needle electrode tip is 30°, with a curvature radius of 15 μm, and the distance between the needle tip and the epoxy resin sheet is 1 mm. The antenna was installed in front of and behind the metal screws on the cable terminal to conduct related PD detection experiments. The oscilloscope used in the experiments was a Tektronix MSO68B model (Tektronix, Beaverton, OR, USA), an 8-channel 10 GHz bandwidth oscilloscope with a maximum sampling rate of 50 GS/s, capable of effectively capturing PD signals. The experimental circuit schematic is shown in Figure 7c, while the corresponding PD experimental platform and the installation setup of the flexible antenna are presented in Figure 7d. Before installation, fixed points for bending were pre-calculated, and the antenna was secured using an ultra-thin PI tape.

### 4.2. Impact of Antenna Bending on PD Detection Results

**Figure 8:**
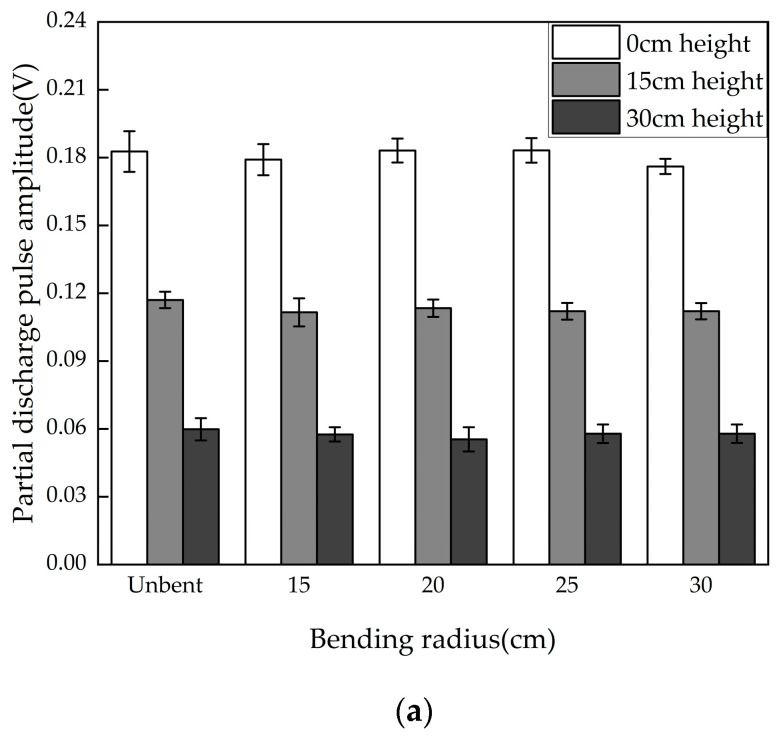

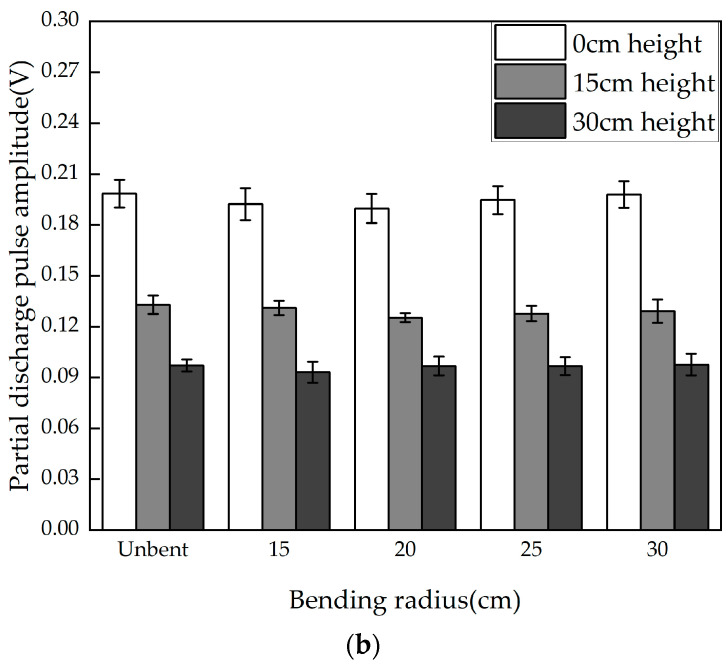
PD pulse amplitude distribution at different bending conditions. (**a**) Metal protrusion defect; (**b**) air gap defect.

The size of the GIS cable terminal varies with different voltage levels. To better install a flexible antenna on the outer wall of the epoxy sleeve for PD detection, the antenna will experience different bending situations during installation. To explore the differences in the detection performance of the flexible antenna in different installation environments and evaluate the generality of the flexible antenna installation, the experiment was set up with five different bending radii: unbent, 15 cm, 20 cm, 25 cm, and 30 cm. Tests were conducted at three different horizontal height differences (the height difference between the center of the antenna and the PD defect). The results are shown in Figure 8.

From the detection results, it can be seen that for the metal protrusion defect, the average PD pulse amplitudes at the three different height differences were approximately 180 mV, 120 mV, and 60 mV. Under the same height difference, the difference between the bending conditions ranged from 3.9% to 7.4%. For the air gap defect, the average pulse amplitudes at the three different height differences were approximately 190 mV, 130 mV, and 100 mV. The differences for various bending conditions at the same height difference were between 4.1% and 5.7%. The overall deviation is relatively small, indicating that the flexible antenna designed in this paper maintains consistent PD detection capabilities under various bending conditions and is minimally affected by bending. The antenna’s bandwidth is also unaffected, with the overall bandwidth variation being less than 1%. This shows that the flexible antenna can maintain stable impedance characteristics in different installation environments.

### 4.3. Influence of PD Source Position on Detection Results

**Figure 9:**
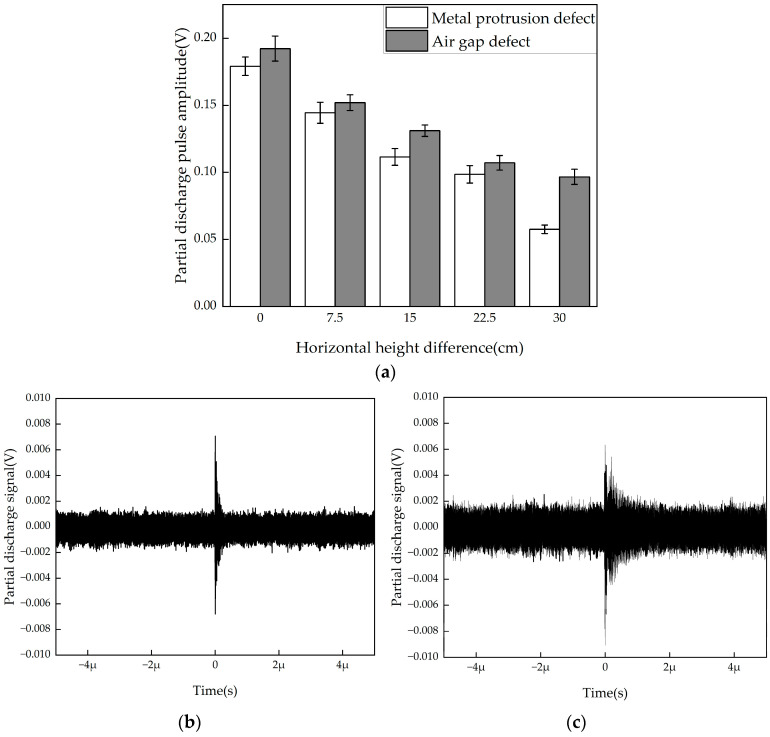
PD experimental results and waveforms at different horizontal height differences. (**a**) PD pulse amplitude at different horizontal height differences; (**b**) metal protrusion defect at 75 cm height PD waveform; (**c**) air gap defect at 75 cm height PD waveform.

PD defects in the GIS cable terminal typically arise from manufacturing or production issues. The position of the PD source is random and cannot be predicted in advance, which can significantly affect the detection results. This section investigates the impact of horizontal height difference between the PD source and the flexible antenna on the detection results. The detection results for the two typical defects are shown in Figure 9a.

The detection results show that, as the horizontal height difference increases, the detected PD pulse amplitude generally decreases. The flexible antenna can effectively reflect the phenomenon of reduced PD electric field strength as the distance increases.

When the distance between the discharge source and the antenna reaches 75 cm, the average PD signal amplitudes for the two defects are 7.04 mV and 5.25 mV, respectively, with waveforms shown in Figure 9b,c. From the waveform, it can be seen that the environmental noise is approximately ±2 mV. The flexible antenna still has good detection capability for weak signals at greater distances. For the GIS cable terminal, the detection range of 75 cm is sufficient to cover the relevant structures, and PD phenomena occurring within the cable terminal can be effectively captured and distinguished from background noise by the flexible antenna designed in this paper.

### 4.4. Influence of Metal Obstruction on PD Detection Results

Due to the compact structure of the GIS cable terminal, the installation and detection of PD antennas are affected by metal obstruction. Metal screws were used to shield the antenna in the experiment, with five different shielding levels: 100%, 75%, 50%, 25%, and 0%. An additional unshielded case was tested, where the antenna was placed between the metal screw and the simulated GIS cable terminal cavity, closely adhering to the outer wall for detection.

**Figure 10:**
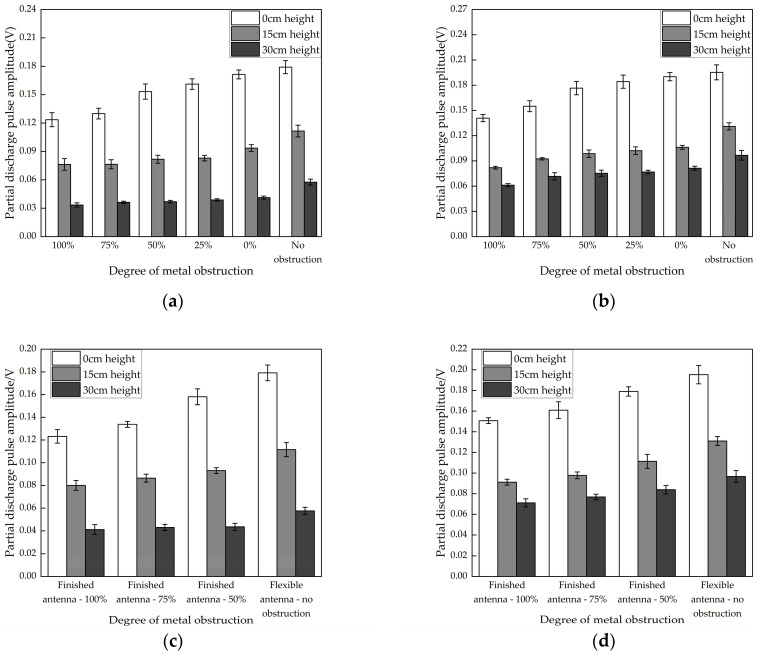
PD detection results under metal obstruction conditions. (**a**) Flexible antenna metal protrusion defect detection results; (**b**) flexible antenna air gap defect detection results; (**c**) detection results for metal protrusion defect with two antennas; (**d**) detection results for air gap defect with two antennas.

The experimental results for two different defects under various metal obstruction conditions are shown in Figure 10a,b. The results indicate that for the metal protrusion defect, compared to the 0% shielding condition, the detected PD pulse amplitudes for the 100% shielding case at three different height differences decreased by 27.9%, 22.7%, and 18.9%, respectively. For the air gap defect, compared to the 0% shielding condition, the pulse amplitudes for the 100% shielding case decreased by 25.8%, 22.8%, and 24.9% at the three different height differences. These comparisons show that metal screws significantly hinder the detection of PD, weakening the detection performance. In cases with weak signals, the PD phenomena might not be detected in time.

In particular, the flexible antenna designed in this paper can avoid the impact of metal obstruction by being directly attached to the outer wall for PD detection. Compared to the unshielded case, with 100% shielding, the detection performance for the metal protrusion defect shows reductions of 31.0%, 31.6%, and 42.0% in the PD pulse amplitudes, respectively; for the air gap defect, the reductions are 27.9%, 37.5%, and 36.8%. When the horizontal height is low, the results for the unshielded and 0% metal obstruction cases are nearly identical, with only the antenna’s distance from the PD source being different. However, as the height difference increases, the presence of metal obstruction, in addition to the metal screw, further weakens the electric field received by the antenna. Therefore, as the PD source moves deeper, the impact of metal obstruction becomes more pronounced.

When comparing the flexible antenna in the unshielded case with the final product antenna under shielding conditions, the results shown in Figure 10c,d indicate that the product antenna performs better, with a 10–20% higher detection amplitude for PD pulses at the same height. However, when the flexible antenna is installed in an unshielded environment, its detection amplitude exceeds that of the product antenna by 10–30% under 50% shielding, demonstrating the advantages of flexible antenna installation and detection capability. The flexible antenna can be more effectively installed in confined metal-shielded spaces and can accurately detect PD phenomena.

## 5. Conclusions

This study addresses the issues of metal obstruction affecting the detection performance of existing GIS cable terminal PD detection and interference in the detection environment. Based on the CPW feeding technology, the grounding plane was extended to the back to suppress environmental interference, and a low-profile directional 3D flexible antenna was designed and fabricated. The following conclusions were drawn from the instrument measurements and PD detection experiments:

(1) The designed antenna has a measured VSWR ≤ 2 in the 0.541–3 GHz frequency range. Both the measurements and experiments show that bending has a minimal effect on antenna performance, and the antenna maintains stable PD detection capabilities under different bending conditions. With a profile of 2 cm, the antenna can be flexibly installed in confined spaces like a GIS cable terminal. The antenna’s radiation pattern indicates directional signal reception, with some suppression of interference from the back;
(2) The flexible antenna still exhibits good detection capability for PD signals when the height difference between the PD source and the antenna is 75 cm. It can clearly distinguish between background noise and PD signals, indicating that the flexible antenna can effectively detect PD phenomena occurring within the overall structure of the GIS cable terminal;
(3) The metal obstruction in the cable terminal weakens the amplitude of the PD signal detected by the antenna by more than 20%. Furthermore, as the PD source moves deeper, the impact of metal obstruction becomes more pronounced. By comparing the flexible antenna designed in this study with existing commercial antennas, the advantages of avoiding metal obstruction for PD detection are verified.

## Figures and Tables

**Table 1:** Parameters of flexible dielectric substrate materials.

Material	*ε_r_*	tan*δ*
PI	3.4	0.008
PDMS	1.68	0.03
PET	4	0.01

**Table 2:** Partial shape parameters of the flexible antenna.

Parameter	Size/mm
*W*	70
*H*_1_	20
*w*	1.4
*a*	53
*b*	69
*c*	32.5
*d*	0.178

**Table 3:** Flexible antenna shape parameters.

Parameter	Size/mm
*W*	70
*H*_0_	197
*H*_1_	20
*w*	1.4
*a*	53
*b*	69
*c*	32.5
*d*	0.178
*e*	2
*l*	1
*r*_1_	13

## Data Availability

Data is contained within the article: the original contributions presented in this study are included in the article. Further inquiries can be directed to the corresponding author.
